# Classifying and Predicting Salinization Level in Arid Area Soil Using a Combination of Chua’s Circuit and Fractional Order Sprott Chaotic System

**DOI:** 10.3390/s19204517

**Published:** 2019-10-17

**Authors:** Anhong Tian, Chengbiao Fu, Xiao-Yi Su, Her-Terng Yau, Heigang Xiong

**Affiliations:** 1 College of Information Engineering, Qujing Normal University, Qujing 655011, China; tianah@mail.qjnu.edu.cn (A.T.); fucb@mail.qjnu.edu.cn (C.F.); 2 Graduate Institute of Automation Technology, National Taipei University of Technology, Taipei 10608, Taiwan; 40125451@gm.student.ncut.edu.tw; 3 Department of Electrical Engineering, National Chin-Yi University of Technology, Taichung 41170, Taiwan; 4 College of Applied Arts and Science, Beijing Union University, Beijing 100083, China; heigang@buu.edu.cn; 5 College of Resource and Environment Sciences, Xinjiang University, Urumqi 830046, China

**Keywords:** fractional order compound master-slave chaotic system, extension matter-element model, arid area soil, dynamic error, salinization level, areas with different levels of human interference

## Abstract

Soil salinization is very complex and its evolution is affected by numerous interacting factors produce strong non-linear characteristics. This is the first time fractional order chaos theory has been applied to soil salinization-level classification to decrease uncertainty in salinization assessment, solve fuzzy problems, and analyze the spectrum chaotic features in soil with different levels of salinization. In this study, typical saline soil spectrum data from different human interference areas in Fukang City (Xinjiang) and salt index test data from an indoor chemical analysis laboratory are used as the base information source. First, we explored the correlation between the spectrum reflectance features of soil with different levels of salinization and chaotic dynamic error and chaotic attractor. We discovered that the chaotic status error in the 0.6 order has the greatest change. The 0.6 order chaotic attractors are used to establish the extension matter-element model. The determination equation is built according to the correspondence between section domain and classic domain range to salinization level. Finally, the salt content from the chemical analysis is substituted into the discriminant equation in the extension matter-element model. Analysis found that the accuracy of the discriminant equation is higher. For areas with no human interference, the extension classification can successfully identify nine out of 10 prediction data, which is a 90% identification accuracy rate. For areas with human interference, the extension classification can successfully identify 10 out of 10 prediction data, which is a success rate of 100%. The innovation in this study is the building of a smart classification model that uses a fractional order chaotic system to inversely calculate soil salinization level. This model can accurately classify salinization level and its predictive results can be used to rapidly calculate the temporal and spatial distribution of salinization in arid area/desert soil.

## 1. Introduction

Chaos refers to irregular, random non-linear phenomena in a certainty system. In reality, it has an inherent regularity, which is a unity of order and disorder. In the natural world, chaotic phenomena are everywhere. To solve actual problems in the natural world, we often build corresponding physical models to find solutions. Integer order differential transformation has been widely applied to soil spectrum-signal processing [1,2,3,4]. However, the physical model it describes is just approximation processing, and needs to use a fractional order differential equation to increase accuracy as the complexity and diversity of the descriptive system increases. Fractional calculus is a generalized form for orders in classic calculus. Compared to traditional calculus, its greatest advantage lies in its memory and inheritance. This makes describing certain physical phenomenons with fractional order differential equation more accurate and effective. Studies have already proven that the fractional order system conforms better to natural law and engineering physical phenomena [5,6,7,8,9,10] and has a more complex dynamic behavior. The development of fractional calculus facilitated fractional order chaotic system research, such as Chua’s circuit, Lorenz system, and the Chen system. When orders become fractional orders, the system still exhibited chaotic phenomenon. In recent years, fractional order chaotic system studies have become popular, and the system has been applied widely to various fields [11,12] such as physics, computer science, ecology, management, biology and medicine.

Soil salinization is the main cause of soil desertification in arid areas. Natural environment and human activities affect the accumulation of soluble salts on the surface, forming different levels of salinization [13,14,15,16,17]. Xinjiang has become an important food and cotton production base for China, as well as being the safety reserve base for food and cotton. Soil salinization seriously threatens agricultural safety in Xinjiang [18,19]. Xinjiang’s arid/desert areas have high evaporation levels and low rainfall. High levels of human activities also have special effects on the moisture and salt status in desert areas. Previously, scholars used soil’s salt spatial and temporal distribution, field measured water/salt migration laws, salt/water content organic physical properties, and image spectrum data to monitor and draw maps in soil salinization studies [20,21,22,23,24]. Nurmemet et al. [25] used salinization areas in the Keriya River Basin in Xinjiang as test areas and used a support vector machine (SVM) to classify soil coverage types in the salinization areas. The cross-validation method was used to ensure optimal classification parameters and a decision tree (DT) solution was applied to increase the accuracy of saline soil estimation. The result showed that an integration of passive reflectance and active microwave remote sensing is an effective way to measure salinization. The DT solution is even more effective and has an accuracy rate of 93.01%. Overall, the study area had a saline soil content of 41.43%. Sarani et al. [26] used the soil in the Sistan plain, Iran, as the research subject. They measured the soil’s physical and chemical properties, then used the data to find the changes in exchangeable sodium percentage (ESP) and sodium adsorption ratio (SAR) in the soil. The result shows that the multilayer perceptron (MLP) is the best neural network prediction model and can accurately estimate ESP and SAR quantity. Vermeulen et al. [27] used extremely high resolution WorldView-2 image to draw soil salt accumulation in South Africa’s Vaalharts region. The result shows that extremely high-resolution images can be used to great effect to detect salt accumulation. Salinity indices threshold based on normalized differences has the best classification effect and has an accuracy of 80%. Xia et al. [28] used a Fourier infrared spectrometer to field measure Xinjiang saline soil. They found that 8000–13,000 nm soil thermal infrared emissivity decreased as the salt content increased and that emissivity spectrum is the most sensitive to salt content at 8000–9500 nm. However, there are insufficient numbers of studies on soil salt content changes and distribution pattern in areas with different levels of human interference. Changes in soil salt content are affected by the natural environment and human activities. To obtain a better classification of salinization level, we need to explore the temporal and spatial change laws in salinization status. Soil salinization exhibits greater temporal and spatial changes, which present a challenge for salinization identification and diagnostic.

Soil salinization is an extremely complex evolution process that is affected by numerous mutually interacting factors. This process exhibits a strong non-linear characteristic [29,30,31]. Temporal and spatial changes in saline soil spectrum are random and uncertain. Traditional mathematical methods have a difficult time accurately describing and analyzing this system change process. Because the conversion function between saline soil spectrum reflectance and soil parameter is a complex non-linear relationship, non-linear science based on chaos theory can explore and analyze this type of non-linear problem [32,33]. However, there are currently few studies on the use of chaotic system to analyze the soil spectrum features of soil with different levels of salinization.

At present, Tian (2019) has proposed the use of fractional-order chaotic system and PNN (probability neural network, PNN) to classify the degree of soil salinization. The classification accuracy is better than the traditional SVM and KNN (K-nearest neighbor, KNN) methods [34], and the simulation results prove that the hyperspectral signals processed by the fractional-order chaotic system are easier to identify, which is beneficial to classification. However, the calculation of PNN is more complicated, the execution rate is slower, and the promotion in practical applications is limited. Because the extension method is simpler than PNN, it is easier to popularize and use. 

Therefore, this paper uses the fractional-order chaotic system to map the soil hyperspectral signal as a chaotic attractor, and the soil salinization classification model is established by using the plane coordinate method and extension theory. The simulation results show that the classification accuracy of the proposed classification method in Area A and B is 90% and 100%, respectively. Compared with other traditional classification methods, the proposed method has shorter calculation time and higher classification accuracy. The expectation is to uncover saline soil change laws for areas with different levels of human interference as well as to provide new thinking for accurately classifying saline soil spectrum data. 

## 2. Theory Introduction

### 2.1. Chua’s Circuit

**Figure 1:**
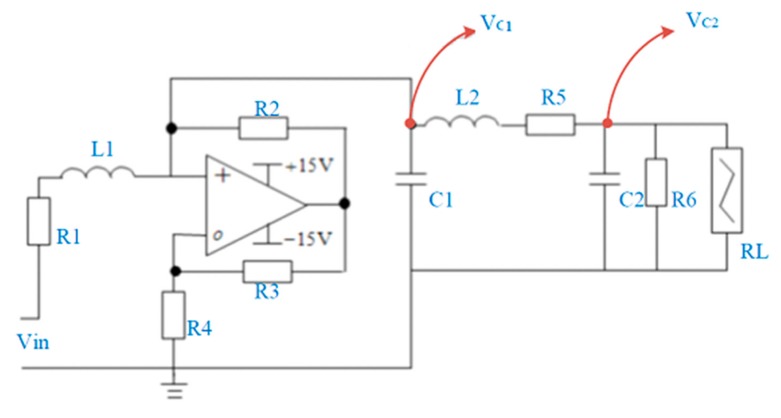
Chua’s circuit.

Chua’s chaotic circuit theory is a research method used in the electronic circuit field to explore a non-linear phenomenon. Chua’s circuit has become the most typical non-linear circuit [35,36]. Architecture based on the Chua’s circuit is simpler compared with other systems. The circuit system can be divided into autonomous and non-autonomous parts. The autonomous circuit does not need an external signal to produce chaotic phenomenon. The non-autonomous part requires the input of an external signal and uses the passing of the time-varying signal through the circuit to produce chaotic phenomenon. This study uses the non-autonomous Chua’s circuit. Figure 1 shows the non-autonomous Chua’s circuit.

**Figure 2:**
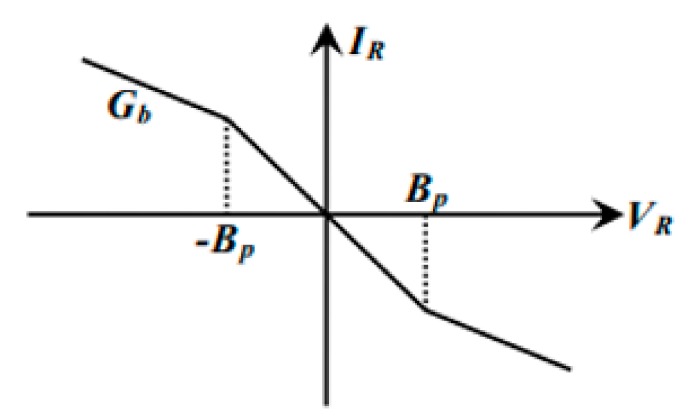
Chua’s circuit voltage-current characteristic curve.

The circuit diagram shows that the non-autonomous Chua’s circuit is composed of the resistor, capacitor, inductor, RL resistor, and operational amplifier. Of which, the RL is the non-linear resistor circuit produced by the operational amplifier that produces different voltage transition in the voltage–current characteristic curve. Figure 2 shows the Chua’s circuit voltage-current characteristic curve. 

Chua’s circuit uses the Kirchhoff’s Law to express the Chua’s circuit into a status equation, as in Equation (1).

(1) $$\left\{\begin{array}{l} C_{1}\frac{dV_{c1}}{dt}=-gV_{C1}+i_{L1}-i_{L2}\\ C_{2}\frac{dV_{C2}}{dt}=i_{L1}-i_{RL}\\ L_{1}\frac{di_{L1}}{dt}=-V_{C1}-i_{L1}R_{1}+V_{in}\sin(2\pi k)\\ L_{2}\frac{di_{L2}}{dt}=V_{C1}+V_{C2}+i_{L2}R_{S} \end{array}\right.$$

In this, *k* is the high order harmonic, $V_{in}$ is this study’s extracted signal, and $i_{RL}$ is defined as Equation (2).

(2) $$i_{RL}=f(V_{c1})=G_{b}V_{C1}+\frac{1}{2}(G_{a}-G_{b})(\left|V_{C1}+B_{P}\right|-(\left|V_{C1}-B_{P}\right|)$$

### 2.2. Fractional Order Sprott Master/Slave Chaotic System

Chaotic systems exist everywhere in nature. Since the mid-1980s until now, chaos theory has been broadly applied to different fields. In the past, engineering has often assumed a linear system for discussion. However, as computing power increases, we have become able to explore and verify the advantages and disadvantages of non-linear systems. Chaos theory allows the non-linear system theory divergence, periodic motion, and aperiodic motion to intertwine under certain parameter conditions. Some notable chaotic systems include Chen-Lee chaotic system, Lorenz chaotic system, and the Sprott chaotic system. In any type of chaotic system a pair of chaotic attractors will appear. The high sensitivity of the chaotic attractors towards signals allows the chaotic system’s dynamic error to produce an ordered but non-periodic motion track using the attractors as the center. This study uses the Sprott master/slave chaotic system, which is composed of the master system (MS) and slave system (SS), as shown in Equations (3) and (4).

(3) $$\dot{X}=AX+f\left(X\right)$$

(4) $$\dot{Y}=AY+f\left(Y\right)+U$$

where, $X\in R^{N}$ and $Y\in R^{N}\;\mathrm{are}\;$ the dynamic vector in which U is the non-linear control component. $f\left(X\right)$ and $f\left(Y\right)$ are the non-linear vectors and *A* is a m×n matrix. When the master/slave system receives a signal and needs to determine system feature changes, then *U* = 0 is set to obtain the status error. 

Today the master/slave chaotic system has a wide variety of applications [37,38,39]. When the chaotic parameters α and β in the Sprott master/slave chaotic system is equal to 2 and 1, the Sprott master/slave chaotic system is guaranteed to produce strange attractors. Its chaotic system dynamic equation is Equation (5). MS is rendered as Equation (6) and SS is rendered as Equation (7).

(5) $$\left\{\begin{array}{c} \frac{dx}{dt}=y\\ \frac{dy}{dt}=z\\ \frac{dz}{dt}=-\alpha z-\beta y+c\left(sign\;x-x\right) \end{array}\right.$$

(6) $$\left\{\begin{array}{c} {\dot{y}}_{1m}=y_{2m}\\ {\dot{y}}_{2m}=y_{3m}\\ {\dot{y}}_{3m}=-ay_{3m}-by_{2m}-1.2y_{1m}+2sign\left(y_{1m}\right) \end{array}\right.$$

(7) $$\left\{\begin{array}{c} {\dot{y}}_{1s}=y_{2s}\\ {\dot{y}}_{2s}=y_{3s}\\ {\dot{y}}_{3s}=-ay_{3s}-by_{2s}-1.2y_{1s}+2sign\left(y_{1s}\right) \end{array}\right.$$

Yau et al. (2016) [40,41,42] proposed a formula that changes the Sprott master/slave chaotic system into a matrix equation, as shown in Equation (8):

(8) $$\left[\begin{array}{c} E_{1}\\ E_{2}\\ E_{3} \end{array}\right]=\left[\begin{array}{ccc} 0 & 1 & 0\\ 0 & 0 & 1\\ -1.2 & -\beta & -\alpha \end{array}\right]\left[\begin{array}{c} e_{1}\\ e_{2}\\ e_{3} \end{array}\right]+2\left[\begin{array}{c} 0\\ 0\\ sign\left(y_{1m}\right)-sign\left(y_{1s}\right) \end{array}\right]$$

According to the method proposed in reference [41], the $e_{1}$ can be omitted. That is, the Sprott master/slave chaotic system’s error status can be changed to a two stage system for expression, as shown in Equation (9):

(9) $$\left[\begin{array}{c} E_{2}\\ E_{3} \end{array}\right]=\left[\begin{array}{cc} 0 & 1\\ -\beta & -\alpha \end{array}\right]\left[\begin{array}{c} e_{2}\\ e_{3} \end{array}\right]=\left[A\right]\tilde{e}$$

This study used the Sprott master/slave chaotic system as the experimental framework to make non-linear features clearer. According to the fractional calculus proposed by Grünwald–Letnikov, the integration of fractional calculus and the Sprott master/slave chaotic system produces the fractional order Sprott master/slave chaotic system. The fractional order’s proximity value proposed by Grünwald–Letnikov can be indicated as Equation (10):

(10) $$D_{e}^{\pm p}e^{m}\approx \frac{\Gamma \left(m+1\right)}{\Gamma \left(m+1\pm p\right)}e^{m\mp p}$$

where, *m* represent any real number, *e* represents the status error, and *p* = 1 represents normally defined slope. The *p* value discussed in fractional value is between 0 and 1. The Sprott master/slave chaotic system’s synchronization status error equation can be written as fractional order Equation (11):

(11) $$\left[\begin{array}{c} E_{2}\left[i\right]\\ E_{3}\left[i\right] \end{array}\right]\approx \left[\begin{array}{cc} 0 & 0\\ -\beta^{\prime } & -\alpha^{\prime } \end{array}\right]\left[\begin{array}{c} e_{2}{\left[i\right]}^{1+p}\\ e_{3}{\left[i\right]}^{1+p} \end{array}\right]$$

### 2.3. Extension Theory

**Figure 3:**
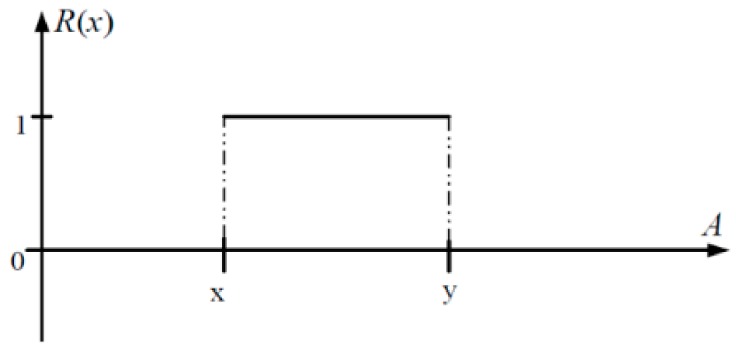
Classic mathematics crisp set.

**Figure 4:**
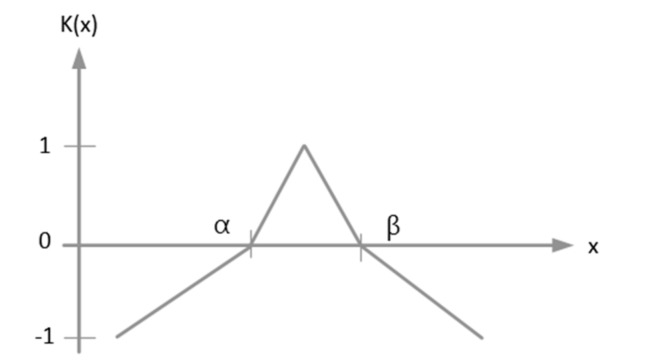
Extension set.

The extension theory was proposed by Wen in 1983 [43]. This theory uses its expansion characteristics to search for regularity between statuses. The objective is to search for a method to solve contradiction problems. It is a type of logical solution analysis that uses mathematical calculation to induce and summarize status feature [44]. Extension theory can be divided into two types of classification, matter-element theory and extension mathematics. Matter-element theory divides an item/event into feature expansion range value and feature value. A matter-element model in expansion theory is used to describe the data of this item/event. Item/event conversion relationship is then used to obtain the proportion of effect produced by each feature. This method can clearly show the importance of a feature to this item/event. Extension mathematics uses extension set and extension correlation function as the indicator to expand the dualism in classic mathematics into <−∞,∞> form. This is used to show that any item/event has its expansion characteristic. Classic mathematics crisp set is shown in Figure 3 and the extension set is shown in Figure 4. 

### 2.4. Matter-Element Model

Extension theory terms everyday things as item/events. To clearly classify the differences of each item/event, each item/event must be given a different name. Each item/event has a different descriptive method. The relationships produced by different patterns and item/event are generally called item/event features and these item/event features have a corresponding feature value. To clearly describe an item/event’s matter-element, three important classifications are required, the name of the item/event, the feature of the item/event, and the feature’s measured value. These three elements are used to produce the matter-element. The matter-element model can be displayed as Equation (12).

(12) $$R=\left(N,c,v\right)$$

where, *R* represents an item/event, *N* is the name of the item/event, *c* is the item/event feature, and *v* item/event is the measured value. Most item/events have multiple features. The vector method can be used to express the matter-element model as Equation (13).

(13) $$R=\left(N,c,v\right)=\left[\begin{array}{c} \begin{array}{ccc} N & c_{1} & v_{1} \end{array}\\ \begin{array}{ccc} & c_{2} & v_{1} \end{array}\\ \begin{array}{cccc} & & \vdots & \vdots \end{array}\\ \begin{array}{ccc} & c_{3} & v_{1} \end{array} \end{array}\right]$$

Based on the *R* feature value in Equation (11) we can build a classic extension domain and section domain. The classic domain and section domain are separately displayed as $V_{d}\;\mathrm{and}\;V_{p}$. The classic domain can be displayed as Equation (14) and the section domain can be displayed as Equation (15). The value of $V_{d}$ is within a numerical value range. $V_{p}$ is the range of the maximum and minimum value of the classic domain’s measured value. In the equation *j* the number of the feature measured value and *i* is the number of the item/event.

(14) $$R=\left(N,c,v\right)=\left[\begin{array}{ccc} N_{i} & c_{1} & v_{1}=\left(a_{i1},b_{i1}\right)\\ & c_{2} & v_{1}=\left(a_{i2},b_{i2}\right)\\ & \vdots & \\ & c_{j} & v_{1}=\left(a_{ij},b_{ij}\right) \end{array}\right]$$

(15) $$R_{p}=\left[\begin{array}{ccc} N & c_{1} & v_{1}=\left(g_{1},h_{1}\right)\\ & c_{2} & v_{1}=\left(g_{2},h_{2}\right)\\ & \vdots & \\ & c_{j} & v_{1}=\left(g_{j},h_{j}\right) \end{array}\right]$$

When the section domain and classic domain is established, we need to calculate the extension distance for later correlation function calculation. The section domain and classic domain’s extension distance is defined as Equations (16) and (17):

(16) $$\rho \left(v,V_{d}\right)=\left|v-\frac{a+b}{2}\right|-\frac{b-a}{2}$$

(17) $$\rho \left(v,V_{p}\right)=\left|v-\frac{g+h}{2}\right|-\frac{h-g}{2}$$

Lastly, we need to calculate the level of extension correlation. Calculation of the correlation level is shown as Equations (18) and (19):

(18) $$D\left(v,V_{d},V_{p}\right)=\left\{\begin{array}{c} \rho \left(v,V_{p}\right)-\rho \left(v,V_{d}\right),V\notin V_{d}\\ -1,\;V\in V_{d} \end{array}\right.$$

(19) $$k\left(v\right)=\frac{\rho \left(v,V_{d}\right)}{D\left(v,V_{d},V_{p}\right)}$$

## 3. Sample Collection

### 3.1. Overview of the Study Area

The research area is located in Xinjiang’s Fukang City. The area’s geographical coordinate is 87°44′ to 88°46′ E and 43°29′ to 45°45′ N, located in the northern foothill of Tarim Basin and the southern edge of the Gurbantünggüt Desert. Fukang is 57 km west of Urumqi and located in the northern foothill of the Tarim Basin economic development belt. Fukang has an agricultural development, industry, and travel industry advantages. The area has temperate continental desert climate with clear seasonal changes. The winter is cold, the summer is hot, and there is very little rainfall (averaging 200 nm annually). The annual air temperature is 6.6 °C and the area is very abundant in thermal energy (average annual sunshine of 2931.3 h). 

### 3.2. Soil Sample Collection

**Figure 5:**
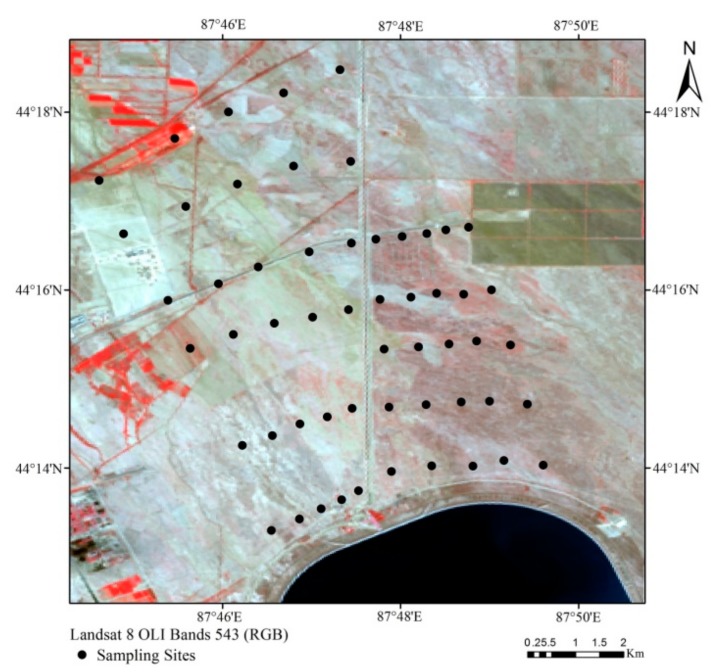
Distribution of soil-sampling points.

Collection of soil samples, gathering of sampling area-related data, and ensuring reasonable sample collection time and route must be prepared ahead of time. A field survey showed that the research area has a giant canal that is 24 m wide at the top, 6 m wide at the bottom, and that is 15.30 km long. This giant canal is used as the boundary to divide the research area into Area A and Area B sampling areas. Area A is located on the right side of the canal and is further away from human activity. This means that the area is less affected by human activities and that the soil has maintained its natural ecological form. Area B is located on the left side of the canal and is closer to Xinjiang’s 102 Production and Construction Army Corp, and is often affected by human interference. This area has been developed into farmland and seedling plantation area. During the soil sample collection process we chose a flat area with no surface vegetation. The choice of sampling points emphasized representativeness and uniformity. This ensures that the samples are representative of the research area’s soil status. The soil samples were field collected in May 2017. The quincunx sampling method was used to collect 0–10 cm of surface soil. Twenty-five points were collected from Area A and 30 points were collected from Area B. The samples were mixed evenly and 1 kg was placed into a sample bag and labeled. Before conducting the field soil sample collection work, a handheld GPS was used to mark the longitude and latitude of each sampling point. After collecting the soil samples the sample was placed in a dry and ventilated laboratory. After natural air drying, the sample was grounded and screened to remove materials other than soil. The soil sample was sent to the Xinjiang Institute of Ecology and Geography where chemical analysis was used to measure the soil salt content. Soil-sampling points are as shown in Figure 5.

### 3.3. Field Spectrum Measurements

While field collecting the soil samples, we also collected field soil spectrums. To field measure the soil spectrums, we used an American ASD FieldSpec3 spectrometer with spectrum wavelength range of 350–2500 nm. For the 350–1050 nm range, an interval of 1.4 nm was used and for the 1000–2500 nm range an interval of 2 nm was used. Resampling interval was 1nm. There were a total of 2151 wavebands. The field spectrum testings were done in the afternoon of clear days. Before each spectrum is measured, the spectrometer undergoes a standard calibration with a standard reference table to reach a nearly 100% baseline. The spectrometer is then pointed towards the ground target to obtain the measure. To make the data representative, five locations around each sampling point is used to collect the spectrum. Each location is measured 10 times. Thus, each soil sample requires 50 measured spectrum curves. The mean of the spectrum reflectance is used as the final result. This can limit the impact on spectrum reflectance caused by soil surface roughness (as a result of soil granules) and ensure spectrum data accuracy.

### 3.4. Spectrum Signal Pre-Processing

Soil spectrum collection is inevitably affected by soil viscosity, particle size, and the environment. Appropriate spectrum pre-treatment can reduce the impact of background noise on the soil spectrum curve. Pre-processing can also increase the correlation between the spectrum and soil content, which can help build a reliable predictive model. The edge bands with lower signal noise (350–399 nm and 2401–2500 nm) and wave bands around the moisture absorption belt (1355–1410 nm and 1820–1942 nm) is removed from the measured spectrum data. Savitzky-Golay convolution smoothing method is used to smooth the remaining spectrum reflectance curves and further remove high-frequency noise interference from the spectrum signal, thereby, increasing the signal–noise ratio.

### 3.5. Soil Salinization-Level Determination Standard

**Figure 6:**
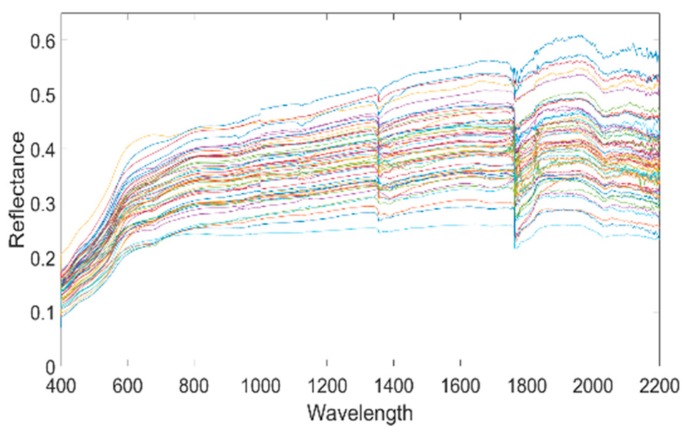
Spectrum reflectance of the 55 sampling points.

**Figure 7:**
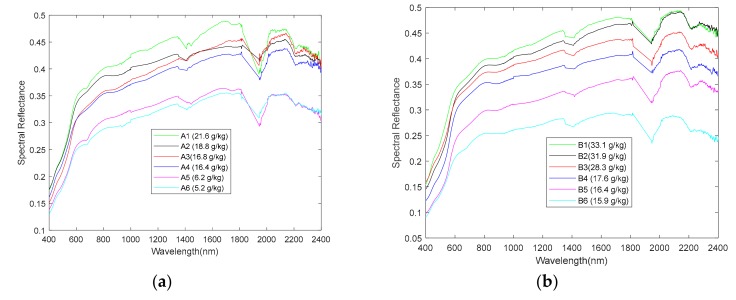
Spectrum reflectance of different salinization level sampling points. (**a**) Area A. (**b**) Area B.

The spectrum reflectance curve of the 55 sampling points in the research area is shown in Figure 6. Figure 6 shows that there is a large quantity of soil spectrum data with a high number of wave band dimensions. As a result, there is an excessively high number of spectrum reflectance information overlap. This is especially true when there are too many soil-sampling points; the spectrum reflectance curve of the sampling points displayed severe overlapping. This makes it difficult to distinguish the salt content of the sampling points and its spectrum reflectance relationship. The soil salinization is classified according to the standard in Table 1 [45]. Six sampling points were selected in Area A, numbered A1 to A6. Six sampling points were selected for Area B, numbered B1 to B6. Figure 7 shows the spectrum reflectance of sampling points with different salinization levels. Figure 7 clearly shows that the higher the soil salt content the greater the spectrum reflectance, and the more severe the salinization level. Conversely, soil with low salt content has low spectrum reflectance.

In addition, the descriptive statistics of Na^+^ and Cl^−^ ion contents are shown in Table 2. The maximum, minimum and mean of Na^+^ ion in Area A is 4.890, 0.640 and 1.590 respectively, and it is 8.299, 0.622 and 2.148 in Area B. The maximum, minimum and mean of Cl^−^ ion in Area A is 9.882, 0.077 and 1.167 respectively, and it is 15.646, 0.077 and 3.081in Area B. So that the Na^+^ and Cl^−^ ion contents in Area B are higher than Area A.

## 4. Simulation Results and Discussion

### 4.1. Fractional Order Compound Master/Slave Chaotic System Motion Track

**Figure 8:**
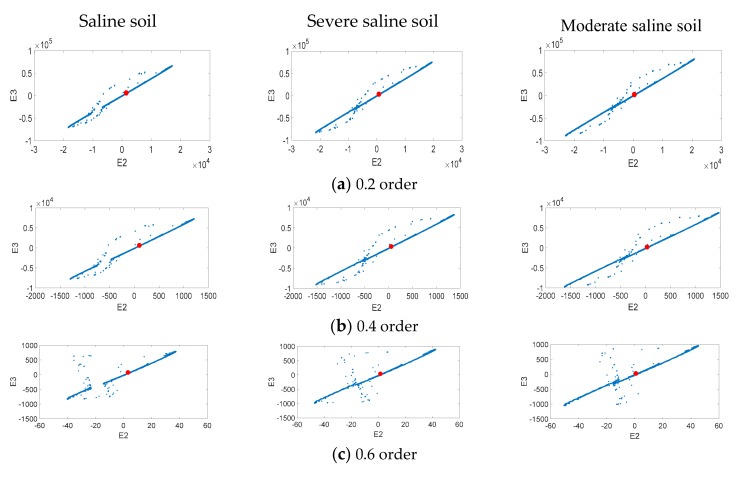

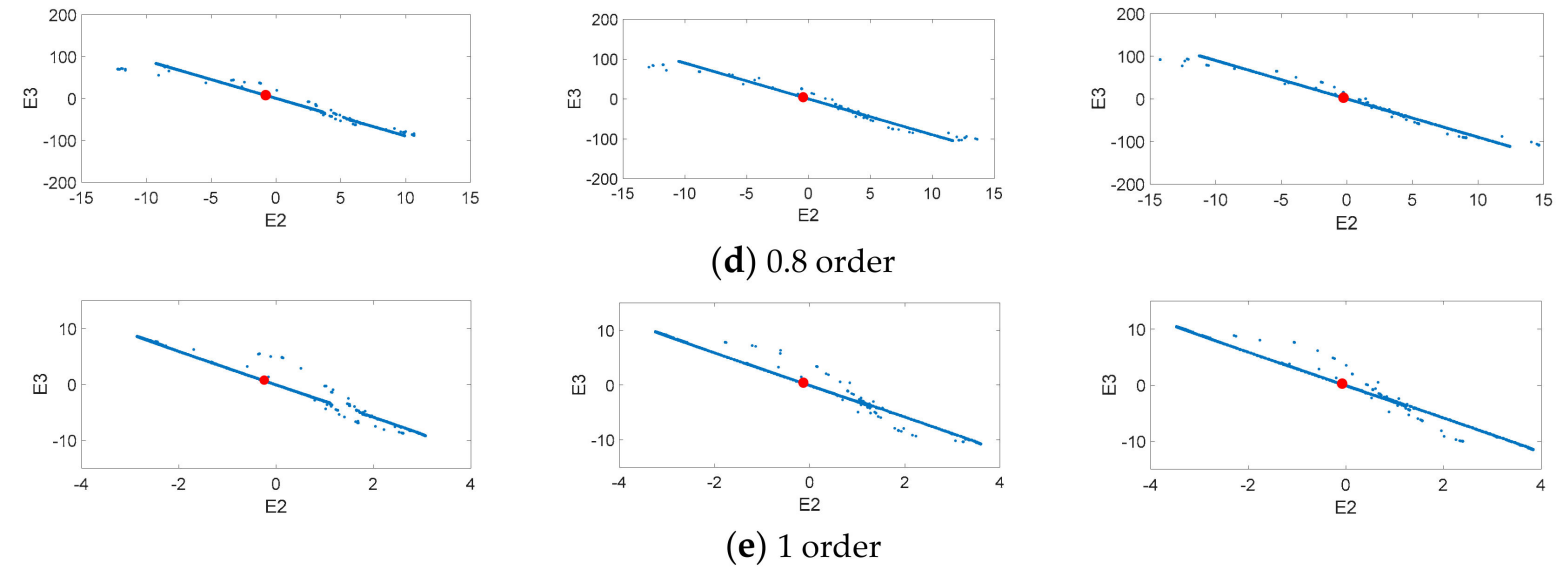
Area A’s chaotic dynamic error distribution.

**Figure 9:**
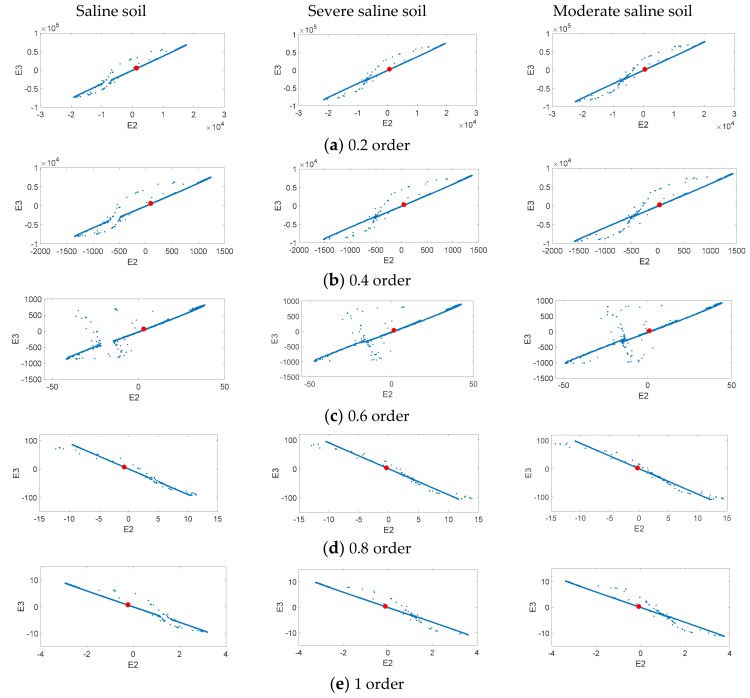
Area B’s chaotic dynamic error distribution.

To find larger fractional order numbers in the dynamic error change at different salinization levels, we used a fractional order compound chaotic system based on the Chua’s circuit and fractional order Sprott master/slave chaotic system as the signal processing tool. For Area A and Area B, we chose one saline soil, one severe level saline soil, and one moderate saline soil sample, and substituted them into the fractional order compound chaotic system to observe the status error changes of different order numbers. To make analysis convenient, 0.2 is used as the order number interval. Five order numbers between 0 order to 1 order (0.2, 0.4, 0.6, 0.8, and 1) were used to observe dynamic error change trends. Figure 8 is the dynamic error distribution of Area A and Figure 9 is the dynamic error distribution of Area B. Figure 8 and Figure 9 simulation results show that the X-axis and Y-axis component change range in moderate saline soil sample dynamic error is greater than that for severe saline soil, and that the dynamic error range of severe saline soil is greater than that of the saline soil sample. Compared to the 0.2 order, 0.4 order, 0.8 order, and 1 order, the dynamic error distribution change of 0.6 order is the greatest. Thus, 0.6 order is most suitable as the follow-up analysis parameter.

### 4.2. Fractional Order Compound Master/Slave Chaotic System Attractors

**Figure 10:**
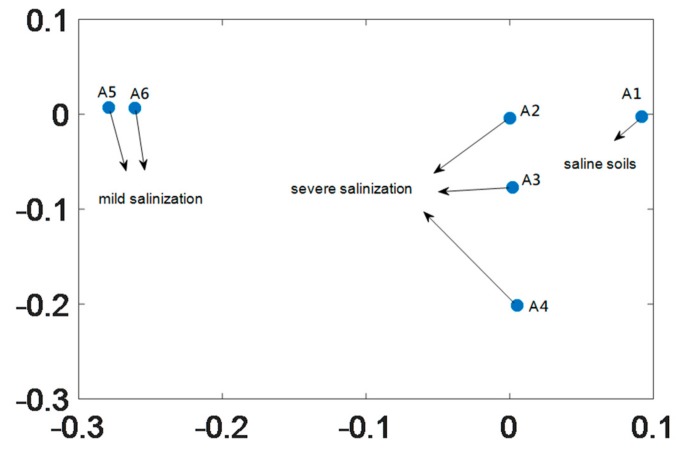
Chaotic attractor distribution for 0.6 order of the six sampling points in Area A.

**Figure 11:**
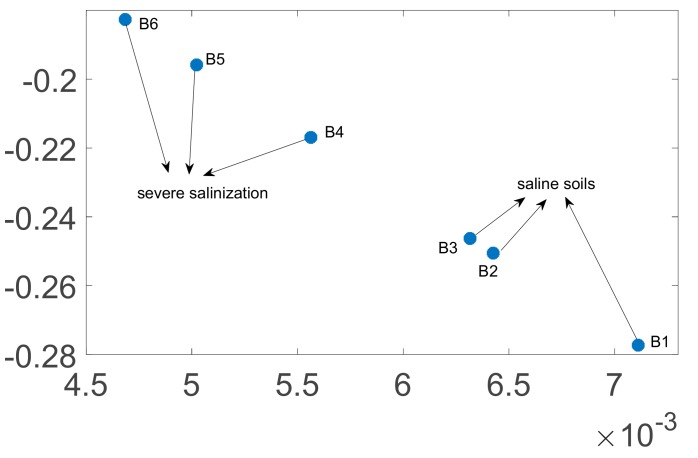
Chaotic attractor distribution for 0.6 order of the six sampling points in Area B.

This study used the extension theory as the back end smart classification method; however, chaotic dynamic error distribution is not convenient for building a classic extension domain. Thus, we chose the fractional order number with the greatest change, and substituted in the master/slave chaotic system to calculate the chaotic attractors. These attractors are used to build the required conditions for the extension classification method. We chose to use the chaotic attractors produced by the fractional 0.6 order compound master/slave chaotic system for the follow-up analysis. The chaotic attractor distribution for Area A and Area B is shown in Figure 10 and Figure 11.

### 4.3. Extension Matter-Element Model Used to Build the Calibration Set

The chaotic attractor coordinate range of soil with different salinization level in Section 4.2 was used to extend the matter-element model. Figure 10 shows that Area A has three salinization statuses, light saline soil, severe saline soil, and saline soil. The vector of the Area A matter-element model is expressed as Equation (20). Because Figure 11 shows that Area B has two salinization statuses, severe saline soil and saline soil, the vector expression for the Area B matter-element model is expressed as Equation (21).

(20) saline soilsc10.09185,−0.00235 c20.09085,−0.00245severe salinizationc10.0051,−0.003 c2[0.0001,−0.2013]mild salinizationc1−0.26, 0.00715 c2−0.279, 0.0067

(21) saline soilsc10.0071,−0.246 c20.0063,−0.277severe salinizationc10.0055,−0.182 c20.0046,−0.216

**Figure 12:**
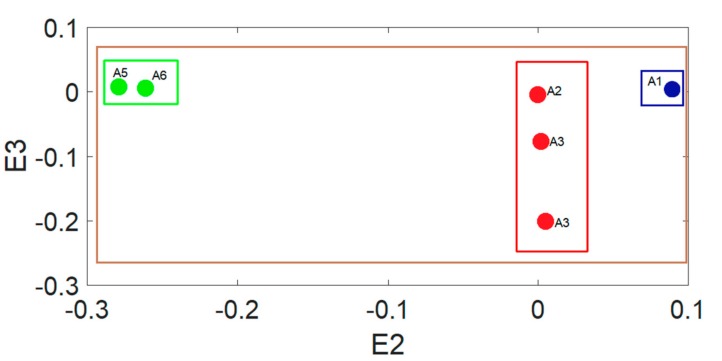
Area A classic domain and section domain.

**Figure 13:**
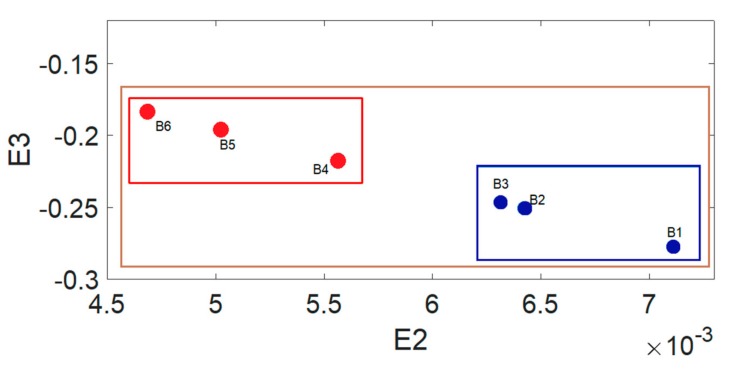
Area B classic domain and section domain.

After the extension matter-element model is built, we can build the extension classic domain based on the matter-element model. The interval range defined by the extension classic domain serves as the basis for the extension system’s determination of the soil salinization. The extension classic domain and section domain for Areas A and B are shown in Figure 12 and Figure 13.

The brown in Figure 12 and Figure 13 indicate the section domain and the blue, red, and green indicates the classic domain of saline soil, severe saline soil, and moderate saline soil, respectively. After building the classic domain and section domain, we can then calculate the extension distance. The extension distance is normalized to obtain the extension correlation level from 1 to −1, which we can use to obtain the sample soil’s salinization level. Table 3 and Table 4 show the extension correlation values of Areas A and B.

### 4.4. The Extension Classification Prediction Results of the Verification Set

Table 5 and Table 6 shows that the extension correlation calculation results of the sampling points match that of the chemical test results. To further verify the classification accuracy of the fractional order chaotic and extension theory system, we picked 10 verification samples from the chemical analysis data from Areas A and B. The Area A verification sample data satisfied the saline soil, severe saline soil, and light saline soil status. The verification data was named Proof A1 to Proof A10. Area B verification sample data matched the saline soil and severe saline soil status. Verification data for Area B was named Proof B1 to Proof B10. Table 4 and Table 5 show the extension correlation calculation results for Areas A and B.

Table 5 and Table 6 show nine of the 10 extension classification data for Area A can be identified, which is an identification accuracy rate of 90%. Because there is less classification status in Area B, 10 of the 10 data can be identified, which is a 100% identification accuracy rate. In the future, large quantity of information can be built into large data bases so that the building of extension classic domain is more accurate, which can improve identification rate and increase system health and applicability.

### 4.5. Comparative Analysis of Classification Method

The classification method proposed in this paper is compared with the traditional classification method. The classification accuracy and calculation time are shown in Table 7.

It can be seen from Table 7 that the fractional-order chaotic system maps the signal as a chaotic attractor. Combining the extension theory with phase plane coordinates can achieve high classification accuracy for the model establishment, which is very suitable for real-time analysis of soil salinization. Compared with other traditional classification methods, the proposed method has shorter calculation time and higher classification accuracy. The classification accuracy of the proposed method is higher than 89%. The classification accuracy of the SVM (Support Vector Machines) and KNN (k-NearestNeighbor) method is about 60%.

The method proposed in this paper mainly uses the fractional-order chaotic system to process the signal. According to the characteristics of the chaotic system which is extremely sensitive to the input signal, the spectral signal can be effectively non-linearly mapped; this not only highlights the slight difference between the states, but also makes the characteristics of the salinization degree state of each soil sample independent and reduces the dimension of the signal. This method is conducive to the subsequent classification research combined with machine learning, extension theory or deep learning to further improve the classification accuracy.

## 5. Conclusions

The soil spectrum feature is a highly complex non-linear system and its spectrum change is affected by topography, parent material, salt content, mineral content, moisture content, organic content, and human activities. Chaos is a unique movement pattern in non-linear dynamic systems. In this study we introduced chaotic techniques to analyze this type of non-linear question. We proposed a classification and prediction method for salinization level in arid area soil based on Chua’s circuit combined with a fractional order Sprott chaotic system. The chaotic dynamic error, chaotic attractor, and the extension matter-element model are used as the classification basis for salinization level. A fractional order chaotic principle and extension theory were combined to build a salinization-level classification model based on a fractional order compound master/slave chaotic system. This model can accurately classify salinization level. In an area with no human interference this model achieved 90% identification accuracy rate. In area with human interference, this model achieved 100% identification accuracy rate. Thus, we were able to achieve smart classification of saline soil spectrum’s non-linear system. This study produced a brand new method for rapidly and accurately testing the soil salinization level in a test area.

## Figures and Tables

**Table 1:** Soil salinization levels.

Value of Salinization Degree	Salt Content (g/kg)
Non salinized soil	Smaller than 5.0
Slightly salinized soil	Between 5.0–10.0
Moderately salinized soil	Between 10.0–15.0
Strongly salinized soil	Between 15.0–20.0
Salinized soil	Greater than 20.0

**Table 2:** Descriptive statistics of Na^+^ and Cl^−^ ion contents (unit: g/kg).

Ion	Area A			Area B		
	Minimum	Maximum	Mean	Minimum	Maximum	Mean
Na^+^	0.640	4.890	1.590	0.622	8.299	2.148
Cl^−^	0.077	9.882	1.167	0.077	15.646	3.081

**Table 3:** Comparison of extension classification correlation value and salt content in Area A’s calibration set.

Sample	Saline Soils	Severe Saline	Mild Saline	Salt Content (g/kg)
A1	1	−1	0.9801	21.6
A2	−0.278	1	−1	18.8
A3	−0.7309	1	−1	16.8
A4	−0.2332	1	−1	16.4
A5	−1	−1	1	6.2
A6	−0.9949	−1	1	5.2

**Table 4:** Comparison of extension classification correlation value and salt content in Area B’s calibration set.

Sample	Saline Soils	Severe Saline	Salt Content (g/kg)
B1	1	−1	33.1
B2	1	−1	31.9
B3	1	−1	28.3
B4	−1	1	17.6
B5	−1	1	16.4
B6	−1	1	15.9

**Table 5:** Comparison of extension classification correlation value and salt content in Area A’s verification set.

Sample	Salt Content (g/kg)	Saline Soils	Severe Saline	Mild Salinization
Proof A1	31.5	1	−1	0.9801
Proof A2	22.7	1	−1	0.9972
Proof A3	22.4	1	−1	0.9867
Proof A4	21.6	1	−0.4193	−1
Proof A5	20.4	1	−1	0.9942
Proof A6	19.0	−0.9	−1	1
Proof A7	18.8	−0.278	1	−1
Proof A8	18.8	−0.708	1	−1
Proof A9	16.8	−0.730	1	−1
Proof A10	16.4	0.9484	1	−1

**Table 6:** Comparison of extension classification correlation value and salt content in Area B’s verification set.

Sample	Salt Content (g/kg)	Saline Soils	Severe Saline
Proof B1	35.7	1	−1
Proof B2	21.0	1	−1
Proof B3	33.1	1	−1
Proof B4	15.9	−1	1
Proof B5	17.1	−1	1
Proof B6	16.4	−1	1
Proof B7	20.4	1	−1
Proof B8	31.9	1	−1
Proof B9	36.4	1	−1
Proof B10	26.0	1	−1

**Table 7:** Comparative analysis of classification methods.

Methods	Classification Accuracy in Area A Verification Set (%)	Classification Accuracy in Area B Verification Set (%)	Total Consumption Time (Second)	Verification Set Consumption Time (Second)
Fractional Sprott Chaos Theory Combined with Extension Theory	90	100	30.8	0.63
Only SVM	60.2	63.9	45.7	1.66
Only KNN	59.4	63.7	45.9	1.64
